# Occupational Physical Hazards and Safety Practices at Dental Clinics

**DOI:** 10.1055/s-0042-1745769

**Published:** 2022-06-21

**Authors:** Abdulaziz Alamri, Mahmoud Fathy ElSharkawy, Dalal Alafandi

**Affiliations:** 1 Department of Preventive Dental Sciences, College of Dentistry, Imam Abdulrahman bin Faisal University, Dammam, Kingdom of Saudi Arabia; 2 Department of Environmental Health, College of Public Health, Imam Abdul Rahman Bin Faisal University, Dammam, Kingdom of Saudi Arabia; 3 Department of Dental Clinics, the Armed Forces Hospitals, Dharan, Saudi Arabia

**Keywords:** noise, radiation, lighting, dental clinic, safety practice, maximum permissible value

## Abstract

**Objective** Worldwide, dentistry is known as a high-level occupational hazard profession. Dental staff is usually exposed to several types of hazards which include chemical agents, physical, psychological stress, and workplace violence, biological and ergonomics. The objectives of this paper were to assess levels of occupational hazards and evaluate safety practices at dental clinics.

**Materials and Methods** At several dental clinics, levels of noise, lighting, and radiation were measured by recommended instruments and the safety practice was evaluated using a validated and reliable questionnaire (during 3 months of 2020).

**Results** The mean levels of noise ranged between 46.3 and 67.2 dB, while the noise dose percent (noise exposure level) ranged between 60.7 and 77.6 dB. The mean levels of lighting ranged from 236.3 lux in the X-ray room to 1,080.3 lux in the dental laboratory. The mean levels of radiation ranged from 7.8 to 12.1 µrem. The mean levels of the three physical hazards were lower than their permissible levels at all locations.

**Conclusion** Levels of noise in dental clinic were affected by the change in the work activities, while this factor has no effect on the levels of lighting and radiation except for certain processes. The demographic variables such as gender, specialization, and the average number of patients showed a significant association with physical hazards, safety practices, while there was no significant association with radiation protection.

## Introduction

Worldwide, dentistry is known as a high-level occupational hazard profession. 1 2 Studies have shown that staff in the dental department are facing worst health problems than other high-risk medical professionals. 3 4 5 Dental staff is usually exposed to several types of hazards which include chemical agents, physical, psychological stress, and workplace violence, biological and ergonomics. 6 7 Noise, radiation, and inadequate lighting are the most important factors causing physical hazards in dental clinics. 8 9

Noise is defined as unwanted sound which results in hearing problems, and its intensity is measured in decibels (dB). 10 Prolonged exposure to noise can cause noise-induced hearing loss, which is defined as bilateral sensorineural hearing loss that develops slowly during various years as the result of exposure to continuous or intermittent loud noise at the workplace. 10 11 Sources of noise in dental clinics include high-speed and low-speed handpieces, high volume suction, ultrasonic instrument, mixing device, and trimmers. 12 13 14 As reported in a study, 16.6% of subjects reported tinnitus, 30% had difficulty in speech discrimination, and 30.8% had speech discrimination due to background noise. 15

In dentistry, a radiograph is mainly used to diagnose and evaluate problem-related to oral diseases and for better treatment planning. Radiographic equipment is commonly used and placed in dental clinics which is considered an important part of the dental assessment. 16 17 Dental staff may expose to both ionizing and nonionizing radiation during dental practice. Nonionizing radiation has become more of interest to dental physicians who are using ultraviolet and blue light to cure or polymerize different types of dental materials. The wavelength's exposure may destroy multiple areas of the eyes, including cornea, lens, and retina. 18 19

The dental staff is exposed to very high luminance for a long time because the dental practice depends on light. Imperfect light plays an important role and adversely influences visual performance which results in visual discomfort with stress-associated and physical effects such as headache, pain, and watering eyes. 20 In case of a mistake during a dental procedure, the adverse impact on the patient will occur, so the visual task of the dental procedure is very critical. 21 The optimal lighting of the oral cavity are usually resulted by a close, frequent, and long operation which results in eye fatigue and eye strain. The light should be distributed uniformly in the dental office and laboratory area to avoid contrast. 22 A spectrophotometer was used to measure the ambient lighting in 32 dental private practices, where it was concluded that the ambient light is not ideal for visual shade matching. 23

Without safety protocols, practices may have prominent negative impacts on the operation in dental clinics. Inadequate workplace design is considered as one of the major causes of accidents. 24 25 More than one study in India have revealed 76 to 77% injuries of the dental staff by the sharps tools, 42 to 43% workplace stress, 40% musculoskeletal disorder, and 24% allergic disease. 9 26 27 Another recent study revealed that more than 20% of the dentists got injured during their work. 28

Awareness regarding the occupational hazards in dental clinics and the implementation of preventive strategies can provide a safe working environment for all dental personnel. 25 29 The safety practices in dental clinics include, for example, installation of a qualified infection prevention program, maintenance of good housekeeping at all work areas, implementation of an education and training program concerning the physical hazards in dental clinics, and adequate usage of the required personal protective equipment. 30 An example of safety practices in dental clinics is the use of protective eyewear as an important means of preventing occupational injury related to the use of dental curing lights and high-speed rotary instruments; as injury from splatters and projectiles including calculus and flying debris during cavity preparation is a common cause of damage to the eyes, the use of protective eyewear should be emphasized. 31

Unfortunately, several previous studies have shown the poor knowledge among dental staff toward the safety practices in their workplaces. 32 33 As an instance, at the Medical University of Warsaw, a study was done by distributing a questionnaire consisting of several multiple-choice questions to 200 dentists, 200 radiographers, 100 dentistry students, and 100 radiographers' students. It was concluded that radiation realization among dentists, radiographers, and students was inappropriate. 34

This study aimed to assess levels of occupational physical hazards and evaluate safety practices at the dental department of the Armed Force Hospital in Dharan in the Eastern Province of Saudi Arabia. This hospital was selected for our study because it is one of the largest military hospitals in the Kingdom of Saudi Arabia (KSA), it has been certified by the Joint Commission International (for evaluation and quality of medical services), and it receives a large number of patients every day in all specialties due to the presence of many advanced devices and instruments. The outcome of the study is important to both dental practitioners and hospitals, especially the dental department, because it may provide insight into the worker's knowledge of physical hazards and safety practices and the level of hazards in the dental department.

## Materials and Methods

### Study Site and Duration

Eleven sites were selected for evaluating physical hazards in the dental department of the Armed Force Hospital in Dharan in the Eastern Province of Saudi Arabia. These sites were divided into five different departments: dental clinics (both general and pediatric dentistry), dental hygiene (DH) clinic, dental laboratory, central sterile service department (CSSD), and the X-ray room. Levels of noise, light, and radiation were simultaneously measured at all selected sites on three different days in a week (Sunday, Tuesday, and Thursday) during the period of 3 months (January–March 2020). On each day, the measurements were conducted at three different periods (8–9 am , 9–10 am, and 10–11 am ). Selection of these days and periods was based on the change in the number of patients and, hence, the work activities inside dental clinic. Nearly 5,022 readings were recorded for noise, lighting, and radiation during the whole period of study at all selected sites.

### Measurement Techniques

Two types of noise were measured in dB, area noise level and the workers' noise exposures (noise dose percent). The area noise means the general noise in a certain workplace where all workers in a place are nearly exposed at the same time to the same level of noise. This type of noise was monitored during this study by the calibrated TES 1352A Sound Level Meter, TES Electrical Electronic Corp. The workers' noise exposure, or the total dose percent, means the measurement of the cumulative noise dose that a worker (dental staff) is exposed to during the day. During this study, this type of noise was measured by the TES-1354 Noise Dosimeter Exposure Time Sound Level. It is a small, programmed device worn on the body as close to the ear as comfortably/conveniently possible. From four departments in dental clinic (the X-ray room was excluded), at least one worker was selected for measuring the total noise dose for 3 hours (8–11 am ) per day. At the end of this period, the recorded noise dose percent was transferred to the corresponding noise exposure level through the following equation:

Timeweighted average (TWA) = 16.61 log (10) (D/100) + 90.

Lighting (or illuminance) was measured by the TES 1337B Lux Intensity Meter, which is an instrument used by hand including a sensor and the measured illuminance is directly displayed in lux (lx). The radiation level (X-rays or any other type) was measured by the RadEye B20-ER Multipurpose Survey Meter from the Thermo Scientific Company. Levels of radiation at the selected locations were measured in microrem. The roentgen equivalent man (or rem) is a unit of equivalent dose, effective dose, and committed dose which are measures of the health effect of low levels of ionizing radiation on the human body. This unit is still used in the United States, while the System International (SI) uses the Sievert unit (Sv). For unit transformation, 1 Sv equals 100 rem.

### Data Collection and Target Population

Evaluation of the safety practice was conducted using a pre-designed questionnaire. It was divided into four parts. The first part represented the demographic data of the applicant and included the general information about age, gender, specialty, and work experience. The second part composed of 11 questions specified for the evaluation of physical hazards handling in dental clinics. The third part included 17 questions specified for the evaluation of the safety practices of the dental staff, such as reporting occupational accidents or health problems, first-aid, warning system, effective fire suppression system, and health care waste management. The fourth part composed of four questions specified for the evaluation of the radiation protection measures. The total number of questions included in the questionnaire was 32. The designed safety questionnaire was reviewed by two experts for its validity and reliability. Confirmatory factor analysis using the structural equation model (SEM) was done to check the validity of the questionnaire.

The questionnaire was distributed among all the dental staff (177 participants) including dentists, dental therapists, dental hygienists, dental assistants, dental laboratory technicians, and CSSD with two exclusion criteria being new staff for less than 1 year and the trainee dental professionals. The response rate for participation was 88.5% of the dental staff, and 62.7% of them were female, while 37.3% were male. The data of the questionnaire were collected through an explanation of its content before giving it to the participants. The collection of the questionnaires from the participants was done through definite time (1 hour to 3 days) based on the availability of and readiness of each participant.

## Statistical Analysis

Results of all measurements and survey processes were analyzed statistically using professional programs such as the Statistical Package for the Social Sciences. For testing the normality of data, we used the Kolmogorov–Smirnov test where most of our data were ranged between −1.96 and +1.96 (through dividing the skewness measure by its standard error). Furthermore, the descriptive statistics and analysis of variance (ANOVA) test were used for comparison of noise, light, and radiation levels, while the chi-square test was used to study the association between demographic variables, physical hazards, radiation protection, and the safety practice of the questionnaire results.

## Results

### Levels of Noise

**Fig. 1:**
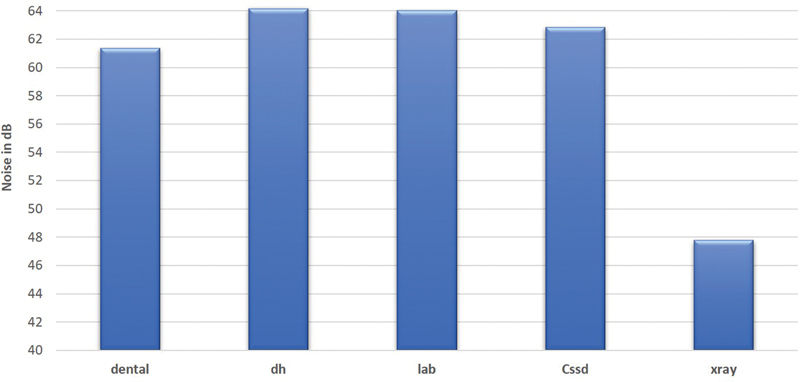
Mean noise levels at different locations in the dental clinics of the Armed Forces Hospital.

**Fig. 2:**
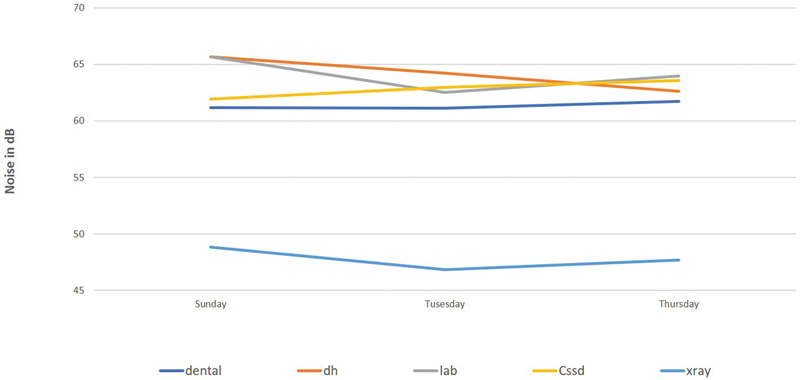
Mean noise levels at different days of the week.

**Fig. 3:**
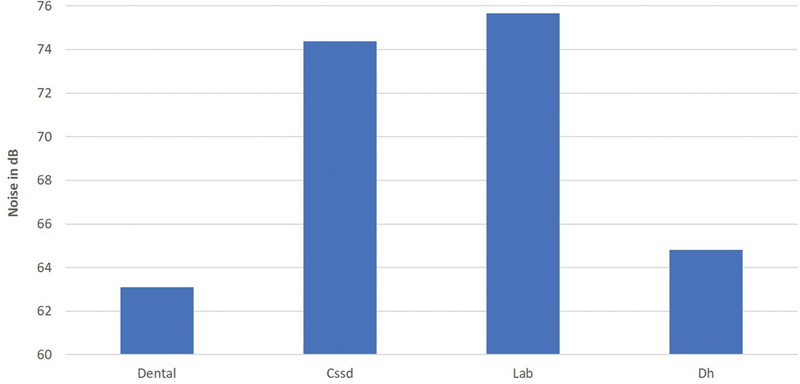
Mean noise dose for workers at different locations in dental clinic of the Armed Forces Hospital.

Fig. 1 represents the mean level of area noise at the five selected departments of dental clinics, while Fig. 2 indicates the mean levels of noise during three selected days (Sunday, Tuesday, and Thursday). The highest mean ± standard deviation (SD) for area noise levels (67.2 ± 11.3 dB) was obtained in the dental laboratory on Sunday during the first period of the day (8–9 am ), while the lowest level (46.3 ± 4.2 dB) was found in the X-ray room on Tuesday during the second period of the day (9–10 am ). The figures illustrate that the highest mean level of noise was found in the dental laboratory and DH clinic, whereas the lowest mean level of noise was found in the X-ray room. Statistically, applying the ANOVA test indicated that there was a significant difference ( *p* < 0.05) between levels of noise in dental clinics and all other four departments. On the contrary, there was a significant difference ( *p* < 0.05) between levels of noise in the X-ray room and other four locations. Additionally, there was no significant difference ( *p* > 0.05) for noise between levels in the DH and laboratory. Levels of noise also varied based on the work time, either for the weekdays or for the day itself. The ANVOA test showed that there was no significant difference ( *p* > 0.05) between levels of noise during the selected three weekdays. Concerning the period of the day, there was no significant difference ( *p* > 0.05) between the first (8.00–9.00 am ) and second periods (9.00–10.00 am ), while there was a significant difference ( *p* < 0.05) between the third one (10.00–11.00) and the other two periods. The mean noise dose percent (noise exposure level) for workers in dental clinics are presented in Fig. 3 , where the dental laboratory workers had the highest exposure level followed by the CSSD workers, while the dental clinics and DH clinics had the lowest levels.

### Levels of Lighting

**Fig. 4:**
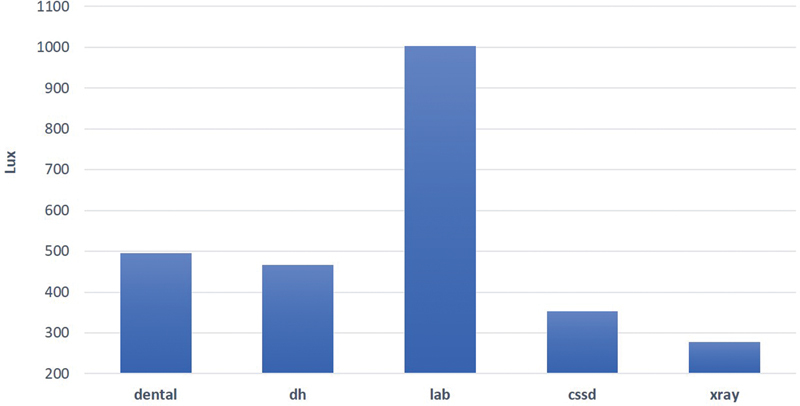
Mean lighting levels at different locations in the clinic of the Armed Forces Hospital.

The highest mean ± SD of lighting levels (1,080.3 ± 464.3 lux) was obtained in the dental laboratory on Tuesday during the second period of the day (9–10 am ), while the lowest one (236.3 ± 115 lux) was found in the X-ray room on Tuesday during the same period. As shown in Fig. 4 , the highest level was found in the dental laboratory, while levels of the other four departments were much lower than the dental laboratory level with significant differences ( *p* < 0.05). It is known that there are different types of work activities in the dental laboratory that need enough and good lightings such as precision work like making dentures, crowns, bridges, and other dental devices. Contrary to noise, lighting levels do not change with the change in activities, except for certain processes such as screening, choosing restorations color shade, cavity preparations, restorations, dental surgeries, and sutures. For this reason, the time factor did not affect the lighting levels through this study and there were no statistically significant differences between levels of lighting during the weekdays or during the day itself.

### Levels of Radiation

**Fig. 5:**
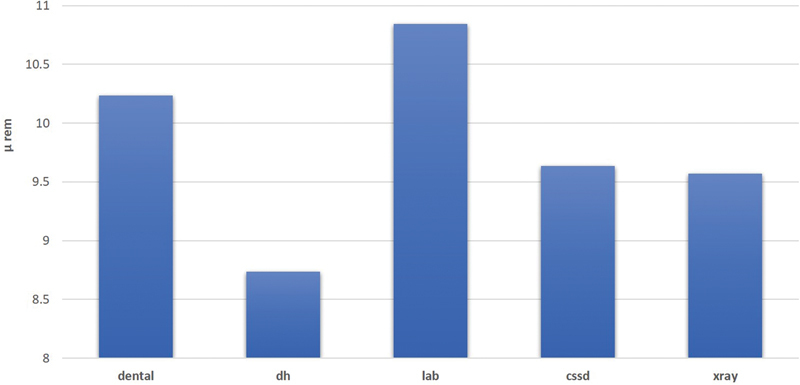
Mean radiation levels at different locations in the dental clinics of the Armed Forces Hospital.

The highest mean ± SD of radiation levels (12.1 ± 3.77 µrem) was also obtained in the dental laboratory on Thursday during the first period of the day (8–9 am ), while the lowest one (7.8 ± 3.3 µ rem) was found in the DH clinic on Thursday between 8 and 9 am. As illustrated in Fig. 5 , the highest level of radiation was found in the dental laboratory followed by dental clinics, whereas the lowest level was found in the dental hygienist clinic. Similar to the lighting levels, the dental laboratory had mean radiation levels higher than the other four locations with statistically significant differences for all locations, while there were no statistically significant differences between levels of radiation during the weekdays or during the day itself.

### Results of the Safety Practice Questionnaire

Of the total number of participants, 62.7% were females, while 37.3% were males. Table 1 presents the responses to questions of the physical hazards in dental clinics. Although the majority of the participants (76.8%) were dealing with an X-ray during their work, most of them were aware neither of how to hold an X-ray film in the patients' mouth while taking a radiograph (74.6%) nor of how to hold a radiographic tube during the exposure (75.1). On the contrary, the majority of participants did not experience hazards such as eye injury and ear fatigue (90.4 and 70.1%, respectively). However, nearly half of the participants experienced annoyance due to loud noise and sharp instrument injury, while the other half did not experience such hazards. About 63% of the participants were not satisfied with the amount of lighting in their workplace. Most of the participants (68.9%) did not frequently use dental loupes for magnification purposes, while 68.4% of them felt as the noise disturbed them in their workplace. Statistically, the demographic variables such as gender, specialization, and an average number of patients showed a significant association with physical hazards ( *p* < 0.05), while the other demographic variables including age, nationality, work experience, and work area did not show any significant association with physical hazards ( *p* > 0.05).

**Table 1:** Responses to questions of the physical hazards

**Physical hazards**		**Frequency**	**Percentage**
Are you dealing with an X-ray during your work?	Yes	136	76.8
	No	41	23.2
Do you hold an X-ray film in the patient's mouth while taking a radiograph?	Yes	45	25.4
	No	132	74.6
Do you hold a radiographic tube during exposure?	Yes	44	24.9
	No	133	75.1
Have you ever experienced any of the following hazards due to your work?			
Eye injury	Yes	17	9.6
	No	160	90.4
Ear Fatigue	Yes	53	29.9
	No	124	70.1
Annoyance due to loud noise	Yes	88	49.7
	No	89	50.3
Sharp instrument injury	Yes	71	40.1
	No	106	59.9
Are you satisfied with the amount of lighting in your workplace?	Yes	66	37.3
	No	111	62.7
Do you use frequently dental loupes for magnification purposes?	Yes	55	31.1
	No	122	68.9
Does the noise disturb you in your workplace?	Yes	121	68.4
	No	56	31.6
Does the noise prevent you from paying attention to your work?	Yes	91	51.4
	No	86	48.6

Table 2 shows the responses to questions of the safety practices for the dental staff. Most of the participants (≥ 80) were aware and adhered to all safety practices. Notably, 42.9% of respondents were not aware of the periodical awareness programs for occupational hazards in the dental clinic they work in. Only (22.6%) were attending a workshop about occupational hazards. Statistically, the demographic variables such as gender, nationality, specialization, and work area demonstrated a significant association with safety practice ( *p* < 0.05). However, other demographic variables such as age, work experience, and an average number of patients did not show a significant association with safety practice ( *p* > 0.05).

**Table 2:** Responses to safety practice

**Safety practices**		**Frequency**	**Percentage**
Ensure instrument sterilization	Yes	167	94.4
	No	10	5.6
Use of protective eyewear	Yes	144	81.4
	No	33	18.6
Use of facemask	Yes	169	95.5
	No	8	4.5
Change gloves between patients	Yes	173	97.7
	No	4	2.3
Wash hands before and after gloving	Yes	146	82.5
	No	31	17.5
Use hands sanitizer agent	Yes	131	74
	No	46	26
Wear protective aprons during work	Yes	144	81.4
	No	33	18.6
Do you know how to maintain a comfortable light in the treatment room?	Yes	111	62.7
	No	66	37.3
Do you always use indirect vision while treating maxillary teeth?	Yes	107	60.5
	No	70	39.5
Is there a system of reporting occupational accidents or health problems in the dental clinic you work in?	Yes	165	93.2
	No	12	6.8
Is there a first-aid system for prompt dealing with any occupational accidents or health problems in the dental clinic you work?	Yes	160	90.4
	No	17	9.6
Is there a warning system for any accident or emergency case in the dental clinic you work?	Yes	161	91
	No	16	9
Is there an effective fire suppression system in the dental clinic you work in?	Yes	168	94.9
	No	9	5.1
Is there a safe system for health care waste management and disposal in the dental clinic you work?	Yes	172	97.2
	No	5	2.8
Is there a periodical awareness program for occupational hazards in the dental clinic you work in?	Yes	101	57.1
	No	76	42.9
Are there safety signs obvious for all staff and visitors in the dental clinic you work?	Yes	168	94.9
	No	9	5.1
Did you attend any workshop about (occupational hazards)?	Yes	40	22.6
	No	137	77.4

Table 3 indicates the responses to questions of the radiation protection measures in the clinic. It is shown that 88.7% of respondents stated that they stand behind a suitable barrier or wall during exposure of the film. About 85.3% of respondents were used to wearing a radiation exposure detection device in the clinic. Besides, 27.1% of respondents failed to follow the position distance rule when not leaving the room/barrier was not used. About 21.5% of respondents did not undergo periodic check-ups performed for their X-ray equipment. Statistically, there was no significant association between all demographic variables and radiation protection ( *p* > 0.05).

**Table 3:** Responses to questions of the radiation protection measures

**Radiation protection**		**Frequency**	**Percentage**
Do you follow the following radiation protection measures in the clinic?			
Stand behind a suitable barrier or wall during exposure of the film.	Yes	157	88.7
	No	20	11.3
Follow the position distance rule when not leaving the room/barrier is not used	Yes	129	72.9
	No	48	27.1
Wear a radiation exposure detection device in the clinic	Yes	151	85.3
	No	26	14.7
Periodic check-ups performed for your X-ray equipment?	Yes	139	78.5
	No	38	21.5

The overall frequency of the risk factors in the dental clinic of Armed Forces Hospital is presented in Table 4 . It is evident that only 26.6% of respondents experienced physical hazards. Most of the respondents (98.3%) were aware and adhered to the safety practices. About 75.7% of respondents followed radiation protection measures in their clinics.

**Table 4:** Overall frequency of the risk factors

**Risk factors**		**Frequency**	**Percentage**
Physical hazards	Yes	47	26.6
	No	130	73.4
Safety practices	Yes	174	98.3
	No	3	1.7
Radiation protection	Yes	134	75.7
	No	43	24.3

## Discussion

Generally, the use of suction, ultrasonic scalar, or high-speed turbine can lead to temporary or permanent hearing loss. 35 It is essential to conduct noise monitoring in dental clinics to create a better working environment and to reduce occupational health effects of noise. The results of this study showed that noise levels differ based on the activity and the location inside dental clinics. For this reason, the highest mean levels of noise were obtained in the dental laboratory followed by the DH clinic, dental clinics, and CSSD, while the lowest mean levels were found in the X-ray room. It is known that there are different types of work activities in the dental laboratory that consider a source of noise such as grinding, trimming, denture-polishing unit, and compressed air. On the contrary, the work activities in the X-ray room is very limited compared with the other departments in dental clinics. On the other hand, levels of noise also vary based on the work time, either for the weekdays or for the day itself, where number of patients differ with time, and consequently, the work activities in dental clinics also change. The National Institute for Occupational Safety and Health 36 recommends a TWA of 85 dBA for an 8-hour work. Also, according to reports from the Occupational Safety and Health Administration, only 8 hours of exposure in a continual way to the noise level of 85 dB is an allowable limit daily. 13 During this study, all mean levels of noise at all departments and sites were lower than the recommended limit (85 dBA). Results of this study are comparable to the results of other studies in different countries of the world. For example, the study which has been done to examine noise exposure and its related problems in 114 students in UAE revealed that the maximum noise levels were around 65 to 79 dB, where the dental laboratory had the highest noise level. 10 Another study was done to measure the noise levels produced by various pieces of dental equipment in a dental institution in India concluded that the noise levels generated varied between 72.6 dB in pre-clinics and 87.2 dB in prosthesis laboratory, where the noise level recorded from the dental laboratory was found to exceed the maximum permissible value of 85 dB. 13 A review study conducted revealed that dental workers are exposed to different levels of noise based on the nature of their work in the laboratory, where they are usually exposed to an occupational hazards in respect to noise-induced hearing loss during their work. 10

Good lighting in the workplace is a very important factor for conducting efficiently the required tasks in dental clinics without harm or accidents. Poor lighting can affect both physically and mentally, with symptoms such as eye strain, headache, and fatigue, as well as stress and anxiety. 37 Similar to noise, lighting levels differ depending on the location and type of work, the highest lighting levels were recorded in the dental laboratory during this study. It is known that there are different types of work activities in the dental laboratory that need enough and good lightings such as precision work like making dentures, crowns, bridges, and other dental devices. Contrary to noise, lighting levels do not change with the change in activities, except for certain processes such as screening, choosing restorations color shade, cavity preparations, restorations, dental surgeries, and sutures. 23 For this reason, the time factor did not show any effect on the lighting levels through this study neither during the weekdays nor during the day itself. Based on the Saudi Arabian Standards Organization, 38 Table 5 represents the standard (required) amount of lighting at different activities in dental clinics. During this study, all mean levels of lighting at all locations were efficient for the required activities at each one. A study was conducted to determine the quantity and quality of the ambient lighting used during visual shade matching in a sample cohort of dentists in private practices, which showed that the ambient light in the majority of dental private practices measured was not ideal for visual shade matching. 39 Another study was done to assess the experience and attitudes for illumination among DH students which revealed that students who are using good illumination have a low risk of developing musculoskeletal disorders, eye strain, and fatigue. 40

**Table 5:** Standard (required) amount of lighting for different activities in dental clinics

**Work activities in dental clinics**	**The required amount of lighting (lux)**
General lighting	500
At the patient	1,000
Operating cavity	5,000
White teeth matching	5,000
Color inspection (laboratories)	1,000
Sterilization room	300
Disinfection rooms	300

Radiation is the transmission of energy through space and matter. There are several forms of radiation, including ionizing and nonionizing. X-rays are the ionizing radiation used extensively in medical and dental practices. Even though they provide useful information and aid in the diagnosis, they also have the potential to cause harmful effects. In dentistry, it is mainly used for diagnostic purposes, and in a dental set-up usually, the practicing dentist exposes, processes, and interprets the radiograph. It is critical to reducing the exposure to the dental personnel and patients to prevent the harmful effects of radiation. Radiation protection measures have been advocated to ameliorate these effects. 41 According to Aravind et al, 42 radiation has emerged as a major occupational hazards, and this is associated with a high amount of damage resulting from exposure to radiation. It is important to conduct radiation monitoring in the dental department to create a better working environment and to reduce the occupational health effect of radiation. The essential goal of radiation safety is to prevent injury from exposure to ionizing radiation. Regulations have been established with the following annual occupational dose equivalent limits for adults who make radiographic exposures during the course of their work 41 : the whole-body (total effective dose equivalent) is 5 rem (or 0.05 Sv), skin and extremities (shallow-dose equivalent) is 50 rem or (0.5 Sv), and the lens of the eye (eye-dose equivalent) is 15 rem (or 0.15 Sv). Each licensed dentist shall conduct X-ray operations so that no individual member of the public will receive more than the maximum radiation dose in any unrestricted area as suggested by the California Dental Association 43 and the International Atomic Energy Agency 44 (100 millirems or 1mSv in a year, or 2 millirems or 0.02mSv in any 1 hour). In our study, all recorded levels of radiation were much lower than these recommended levels. A study about occupational radiation procedures and doses was conducted in South Korean dentists 16 revealing that the average annual effective radiation dose was 0.17 mSv and the cumulative effective dose was 0.95 mSv, among 465 monitored dentists, which is consistent to large extent with the results of our study.

Dentists are generally exposed to several occupational hazards during dental practice including infectious hazards due to the risk of exposure to various microorganisms and external factors during the dental procedure, such as a needle injury resulting from anesthetic delivery or a patient's accidental bite. Also, the indirect infection can be contracted through saliva, natural organic dust particles, or gingival fluid. Allergic reactions from latex-containing gloves are a significant cause of skin allergies. Chemicals from toothpaste, detergents, lubricants, solvents, and X-ray products represent chemical hazards. Poor lighting can cause eye fatigue and pain. 45

In our present study, most of the participants are generally aware of the safety practices during dental practice. Wearing protective equipment is the standard behavior for nearly all respondents. This is in agreement with the results of several previous local, regional, and international studies. For example, a study conducted in Saudi Arabia revealed that 96 to 98% of dental professionals were wearing gloves when treating patients. 46 Morris et al 47 showed that approximately 90% of dentists in Kuwait wore gloves, 75% wore masks, and 52% wore eyeglasses. A study was conducted for the Belgian dentists showed that 84.61% were always wearing the lead apron while only 12% of the dentists wore the lead apron while operating an X-ray unit. 48 In the Irish study, 42.0% of dentists wore gloves, 64.8% wore masks, and 66.4% wore eye protection. 49 A similar study in Canada concluded that 91.8% of dentists in Ontario always wore gloves, 74.8% always wore masks, and 83.6% always wore eye protection. 50 On the other hand, our results are not following similar studies in the same field. For example, a study which was done among dental students in Croatia showed that dental students adhere to the School of Dental Medicine with a very low level of awareness of the health hazards of the dental profession. 51 Another survey conducted in Bengaluru among practicing dentists revealed that radiation protection awareness was very low and the necessary measures taken to reduce the exposure were not adequate. 41

Concerning the safety system and waste management in dental clinics, more than 95% of the respondents of our study were satisfied and fully aware of the safety signs and precautions in dental clinics. This percent is excellent compared with the results of other similar studies. A recent study done for 300 dentists from different dental clinics in several regions of Saudi Arabia revealed that 76% of the studied clinics had appropriate safety systems and health care waste management programs, while only 46% had a reporting system for occupational accidents. 26 A cross-sectional study done for 525 dentists in the Western Indian private dental practitioners revealed that nearly 95% ensured sterilization of the instruments, 61% were used protective eyewear, 72% used face masks, 88% washed their hands before and after gloving, and 53% wore an apron during the working hours. 30 In India, an investigation among Navy dentists revealed that 47% of them experienced an injury from a sharp instrument during 6 months. 52

The interest and response of the dental staff to attend any workshop about occupational hazards is still weak. In our current study, only 22.6% of the respondents participated in such workshops. Unfortunately, it is the case with most of the dental staff as reported in several studies. A cross-sectional questionnaire-based survey conducted among 300 dentists of both private and governmental dental clinics in Saudi Arabia showed that only 24% attended the workshops on occupational hazards, which is in full accordance with our results. 26 Similarly, only 29.41% of the dental surgeons of the Indian Navy previously attended a workshop on occupational hazards. 51 One of the main conclusions of several studies in dental clinics was the importance of regular workshops, seminars, and educational programs on occupational hazards for all clinical dental staff to update their knowledge about various workplace hazards and the standard safety practices to avoid it. 53 54

Recently, several studies were conducted during coronavirus disease 2019 (COVID-19) to assess the safety awareness between the dental care patients and dentists. 55 For example, a study was conducted to evaluate the patient's knowledge, attitude, and practice of cross-infection control in dentistry during COVID at Altamash Institute of Dental Medicine, Karachi, where the majority of the participants agreed that the proper sterilization of instruments and disinfection of dental operatory are necessary and a large number of participants also agreed that proper disposal of waste is of utmost importance for cross-infection control. 56 In Pakistan, a pilot-tested questionnaire was sent to dental professionals through an online link to assess the knowledge, attitudes, and clinical practices of dental professionals regarding the prevention and control of COVID-19. This study revealed that dental professionals had adequate knowledge about COVID-19, but a few of them were comfortable in treating patients during the pandemic. 57 In Italy, an online questionnaire was submitted to the Italian population to investigate the effect of the COVID-19 pandemic on people's mental and physical balance, oral hygiene habits, type of diet, perceived safety of returning to the dentist, and aesthetics with the use of masks, and it was found that 72% of participants were not concerned about returning to the dentist and 75% of the participants felt that the mask did not diminish the beauty of their smile. 58 Another web-based survey was performed using Google forms questionnaire sent to dentists in Brazil, where a higher percentage of dentists from the least-affected states continued routine dental treatments; dentists were younger and presented a significantly lower level of concern about dental treatments and oral health conditions of their patients. 59

## Conclusion

It is essential to conduct noise monitoring in the dental department to create a better working environment and to reduce occupational health effects of noise. Results of this study showed that noise levels differ based on the activity and the location. However, all mean levels of noise at all sites inside the dental clinics of Armed Forces Hospital in Dharan were lower than the recommended limit (85 dBA). The lighting and radiation levels differ depending on the location and type of work. Contrary to noise, the lighting and radiation levels do change with the change in activities. Appropriate restriction of personnel and the public from areas where radiation is used must be considered by the designation of “controlled areas”, practically in dental radiography outside of the primary X-ray beam and at least 1.5 m away from the X-ray tube or patient in any other direction. Results of this survey revealed that staff in the dental clinics of Armed Forces Hospital have generally excellent and proper knowledge for the protective measures which enable most of them to avoid the health problems or hazards in their workplace. However, dental workers should upgrade their knowledge by participating in continuing dental education.

## Limitation of the Study

Although this study was conducted in an important and a representative hospital in KSA, the data are still representative of a limited number of clinics compared with the large number of such clinics in KSA. However, this work is believed to create an incentive for its repetition in most similar clinics.

## Highlights

This study was conducted to assess levels of noise, lighting, and radiation in the dental department of the Armed Forces Hospital in Dhahran, KSA, and evaluate safety practices and awareness among its staff. Nearly all staff of the department participated as respondents. Results of this study revealed that staff of the Armed Forced dental clinic has generally excellent and proper knowledge of the protective measures which enable most of them to avoid the health problems or hazards in their workplace.
